# Changes in water content and distribution in *Quercus ilex *leaves during progressive drought assessed by *in vivo *^1^H magnetic resonance imaging

**DOI:** 10.1186/1471-2229-10-188

**Published:** 2010-08-24

**Authors:** Jordi Sardans, Josep Peñuelas, Silvia Lope-Piedrafita

**Affiliations:** 1 Unitat d'Ecofisiologia i Canvi Global CSIC-CEAB-CREAF, CREAF (Centre de Recerca Ecològica i Aplicacions Forestals), Universitat Autònoma de Barcelona, E-08193 Bellaterra, Spain; 2 Servei de Ressonància Magnètica Nuclear, Universitat Autònoma de Barcelona, E-08193 Bellaterra, Spain

## Abstract

**Background:**

Drought is a common stressor in many regions of the world and current climatic global circulation models predict further increases in warming and drought in the coming decades in several of these regions, such as the Mediterranean basin. The changes in leaf water content, distribution and dynamics in plant tissues under different soil water availabilities are not well known. In order to fill this gap, in the present report we describe our study withholding the irrigation of the seedlings of *Quercus ilex*, the dominant tree species in the evergreen forests of many areas of the Mediterranean Basin. We have monitored the gradual changes in water content in the different leaf areas, *in vivo *and non-invasively, by ^1^H magnetic resonance imaging (MRI) using proton density weighted (ρ_w_) images and spin-spin relaxation time (T_2_) maps.

**Results:**

ρ_w _images showed that the distal leaf area lost water faster than the basal area and that after four weeks of similar losses, the water reduction was greater in leaf veins than in leaf parenchyma areas and also in distal than in basal leaf area. There was a similar tendency in all different areas and tissues, of increasing T_2 _values during the drought period. This indicates an increase in the dynamics of free water, suggesting a decrease of cell membranes permeability.

**Conclusions:**

The results indicate a non homogeneous leaf response to stress with a differentiated capacity to mobilize water between its different parts and tissues. This study shows that the MRI technique can be a useful tool to follow non-intrusively the *in vivo *water content changes in the different parts of the leaves during drought stress. It opens up new possibilities to better characterize the associated physiological changes and provides important information about the different responses of the different leaf areas what should be taken into account when conducting physiological and metabolic drought stress studies in different parts of the leaves during drought stress.

## Background

Changes in water content in the different leaf parts in response to progressive drought determine leaf tissue functioning, yet these dynamics are not well known. Moreover, a better understanding of water loss would improve the criteria as to what parts of the leaf are the most appropriate for conducting physiological measurements during the process of drought stress. MRI (Magnetic Resonance Imaging) is a good candidate for performing *in vivo *non intrusive measurements of leaf water content, distribution and dynamics in leaves.

In standard MRI of living systems, signal intensity (S) comes from ^1^H nuclei of free water and depends on a number of factors. When using a spin-echo pulse sequence, signal intensity is described as [1]:

(1) $$S\propto \rho \;\left[1-e^{-TR/T_{1}}\right]\;e^{-\;TE/T_{2}}$$

In this expression, there are two instrumental parameters: TE and TR. TE, the echo time, is the time between the 90° radiofrequency excitation pulse and the formation of the spin-echo signal. TR, the repetition time, is the recovery time between pulse train repetitions. The other three parameters, ρ, T_1_, and T_2_, are sample-intrinsic parameters describing the proton spin density, the spin-lattice relaxation time and the spin-spin relaxation time, respectively. As can be seen from eq. 1, image contrast can be manipulated by using the appropriate experimental parameters. For example, image contrast will mainly depend on proton density when choosing a long TR and a short TE. Images obtained in this way are referred to as proton density weighted (ρ_w_) images and may be seen as topological representations of the mobile water fractions in soft-tissue specimens. ρ_w _image intensity is equivalent to the number of hydrogen protons in the sampled volume. There are several factors that influence T_1 _and T_2_, and therefore the MRI signal intensity, as for example solution composition, solution concentration, pH, viscosity, and cell structure [2]. As the signal intensity is a result of many protons acting coherently, it decays as the proton population loses coherence. The time in which this occurs, T_2_, depends on the molecular tissue environment surrounding the water molecules, in particular on the proportion of water molecules that solvate different polar cellular molecules relative to the free water molecules. Thus, measuring T_2 _provides information related to the dynamics and the molecular environment of water molecules at sub-milimolar resolution. By determining T_2 _coupled with quantitative proton density imaging [3], it is possible to compile a detailed description of how water density and the molecular environment around water change *in vivo *with time without destroying the sample in the process.

MRI has already been used for non-invasive studies of physiological and ecophysiological processes in plants during the last two decades in different plant tissues [4-6]. This technique has been shown to be useful in order to study water content in different plant tissues [7-9]. In addition, MRI studies have been used to study plant physiological responses to several stress types, such as drought [10]. Of the different plant organs and tissues, leaf has been noticeably less studied by MRI than the other plant organs, in spite of previous studies that have proved that NMR is suitable to study leaf water content [11]. Nuclear magnetic resonance spin-lattice relaxation time (T_1_) has proved to be useful to determine water exchange time and permeability coefficient of membranes during storing after harvest [12] and to study chloroplast membrane permeability [13]. Proton spin-spin relaxation time (T_2_) has been successfully used to detect water compartment in wheat leaves [14]. In this line, 2D NMR imaging has proved to be suitable to determine the spatial distribution of chloroplast in leaf cells [15]. The use of T_2 _to investigate leaf mobility in leaves submitted to different temperatures has revealed the existence of two different water fractions in cells, bound and free bulk water with distinct relaxation times [16]. T_2 _also increases with the membrane injury produced when temperature rises up to 39-40°C [16,17]. Recently, Capitani *et al*. [18] using a portable unilateral NMR instrument in the study of a slowly developing moderate drought stress in the field and the laboratory observed significant relationships between NMR signals and leaf water content and leaf transpiration. The authors reported a decrease of spin echo intensity and an increase in T_2 _during drought in the crop plants *Phaseolus vulgaris *and *Zea mays*, in the shrub *Cistus incatus *and in the tree *Populus nigra*. In contrast, at the studied levels of drought, the relative water content and NMR signals did not change in *Quercus ilex *leaves [18]. The authors stated that this was due to the large amount of water compartmentalized in cellular structures and macromolecules in this latter species. In another study using MRI to study plant tissues vitrification, Gribble *et al*. [19] reported greater water content in vascular bundles than in the parenchymatic areas. But the changes in leaf water contents during severe drought and the possible different effects in different tissues and leaf areas remain to be investigated. Leaves are not a homogeneous organ and the possibility of asymmetrical response throughout leaf tissues or leaf parts during drought events have not been investigated. Thus, the changes in leaf water contents during severe drought and not only during moderate drought and the possible different effects of drought in different tissues and leaf areas merit to be studied.

MRI may suppose an improvement over other methods used in leaf water content determination. Some of the currently used methods, such as e.g. the gravimetric determination of relative water content (RWC), are intrusive and involve the leaf death, which implies a serious inconvenient for ecophysiological studies. On the other hand, most present plant water status techniques, such as those that measuring hydraulic resistance or water conductance, concentrate in measure either the energy status of water or the water content of the whole plant. Other non intrusive methods, such as those based on leaf dielectric constant [20] or those based on the water capacity to absorb terahertz frequency [21], are promising and certainly non-destructive but have low capacity to distinguish among different leaf areas or tissues. Thus, the possibility to use modern MRI spectrometers with adequate imaging treatment programs allows a non-destructive study of leaf water in different leaf areas and tissues with high resolution and this way allows to conduct an study of spatio-temporal dynamics of leaf water loss during drought. Moreover, coupling proton density weighted images with T_2 _measurements should permit to gain information of both leaf water content changes and water mobility changes in total and different leaf area.

The sclerophyllous tree, *Quercus ilex*, is the dominant tree species in the evergreen forests of many areas of the Mediterranean Basin [22]. The leaves of this species have a great capacity to resist severe drought periods, as an adaptative trait to resist the summer drought period typical of the Mediterranean climate. In this study, we used this species because of its great abundance and because it presents a standard leaf type regarding form and size. The leaf physiology and anatomy, and the drought resistance capacity of this species have been studied from different points of view, such as the leaf morphological plasticity [23], elemental composition and stoichiometry [24-26] photosynthetic capacity [27,28] and transpiration [27,29]. Nevertheless, to date, the leaf water content, distribution, and mobility during progressive drought, i.e. during a continuing decrease in soil water availability, has only been studied under moderate water stress and at whole leaf levels without distinguishing the possible different responses in different leaf parts [18].

In this study we aimed to fill this gap in knowledge by conducting an *in vivo *MRI study on attached leaves of *Quercus ilex *seedlings submitted to water deprivation until death. We followed the spatio-temporal dynamics of the changes in water content in the whole leaf and in the different leaf parts by obtaining quantitative ^1^H density weighted images and T_2 _maps.

## Results

### ^1^H density weighted images

**Figure 1:**
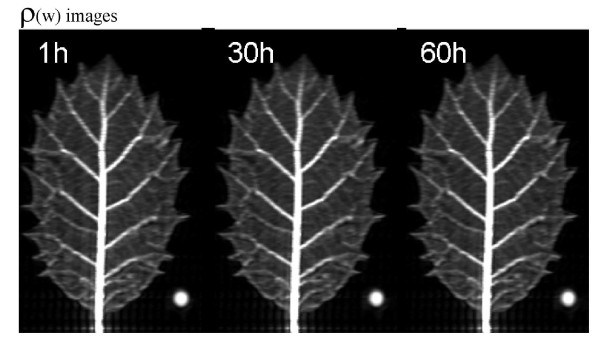
**^1^H density weighted images (ρ_w_) of a *Quercus ilex *leaf at different hours (First hour, 30 hours and 60 hours) after water deprivation**. White circle is the phantom.

**Figure 2:**
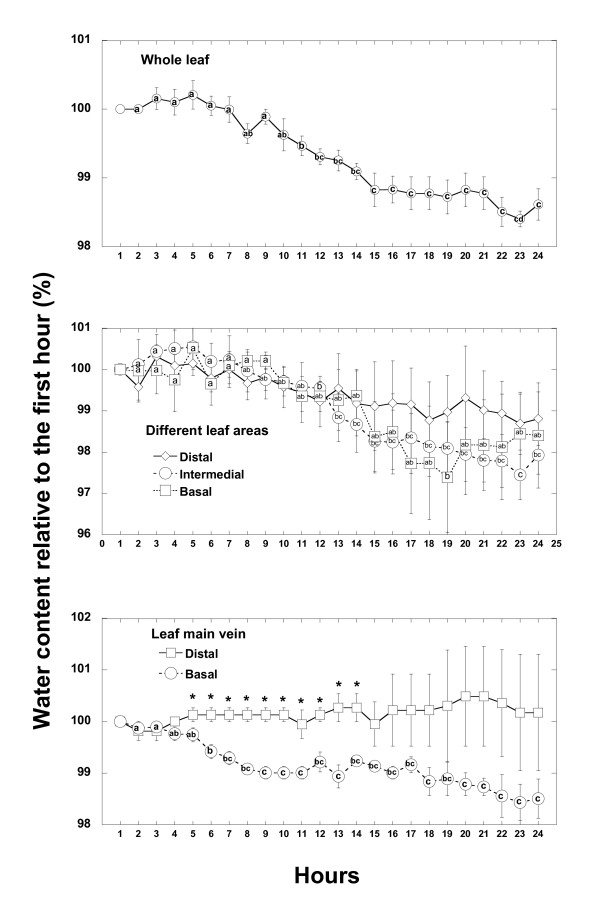
**Time course evolution of water content (relative to the initial) of the different parts of *Quercus ilex *leaves (n = 4) in the first 24 hours (first 12 without light and the last 12 with light) of watering withdrawal**. The same letter means non significant differences, whereas different letters mean significant differences (p < 0.05) at different monitoring time.

During the first 24 hours immediately after the last irrigation, although not apparent by simple image inspection (Figure 1), signal intensity decreased significantly at the whole leaf level (Figure 2a). The great variability of the ρ_w _image intensities did not permit the detection of significant differences among distal, intermediate and proximal leaf areas (Figure 1 and Figure 2b). However, unexpectedly the distal main leaf vein hardly lost water, whereas significant small losses were found in the proximal vein (Figure 2c). Similar results were found in the successive 25-48 hours (data not shown). The hourly changes in the intensity of the ρ_w _images in parenchyma tissues disappeared after two weeks of water deprivation (data not shown).

**Figure 3:**
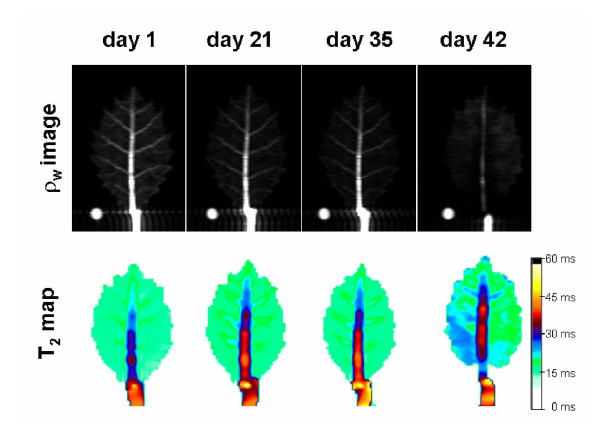
**^1^H density weighted images (ρ_w_) and T_2 _maps of a *Quercus ilex *leaf at different days after withholding watering**. White circle is the phantom.

**Figure 4:**
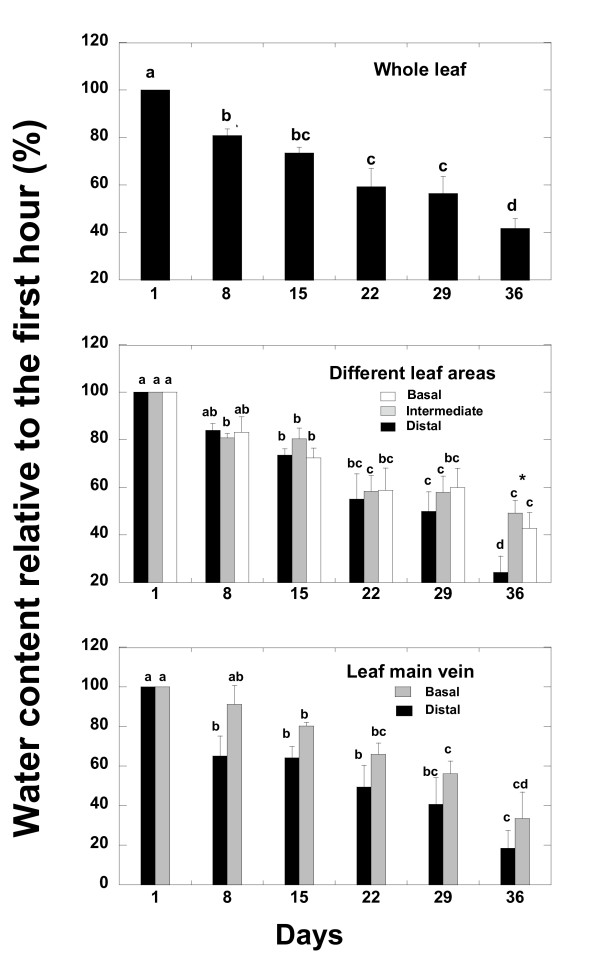
**Time course evolution of water content (relative to the initial) of the different parts of *Quercus ilex *leaves during the first five weeks of the water deprivation**. The same letter means non significant differences, whereas different letters mean significant differences (p < 0.05) at different monitoring time.

**Figure 5:**
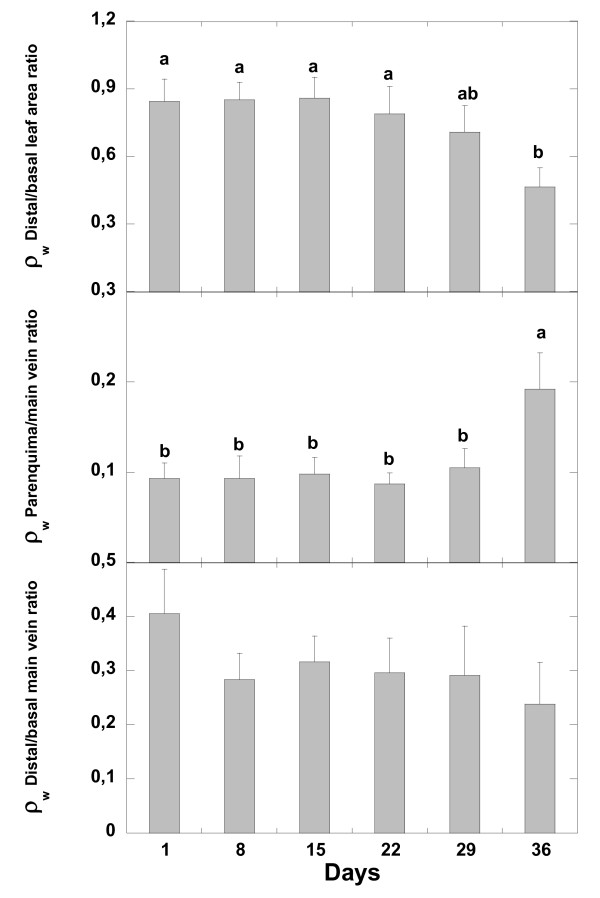
**Time course evolution of the ratios between the absolute ρw values of different leaf areas during the first five weeks of withholding watering**. The same letter means non significant differences, whereas different letters mean significant differences (p < 0.05) at different monitoring time.

In the successive weeks, ρ_w _image intensity decreased at the whole leaf level and in the different leaf tissues (parenchyma and main leaf vein) and leaf areas (Figure 3, 4 and 5). The greatest changes in leaf ρ_w _image intensity occurred during the first and during the fifth week of withholding irrigation. This last week coincided with the appearance of clear necrosis symptoms. The water loss occurred faster in the distal leaf area than in the basal leaf area, mainly between the fourth and the fifth week after watering withdrawal (Figure 3, 4 and 5). The ρ_w _image intensity decreased more in main leaf vein than in parenchyma also in the fifth week of water withholding (Figure 5). This indicates a faster loss of water in main leaf vein than in parenchyma tissues. The distal part of the main leaf vein tended to lose more water than the proximal part (Figure 5). When water was no longer apparent in any tissue of the leaf (six week, Figure 3), it was still abundant in the stem holding the leaf.

**Figure 6:**
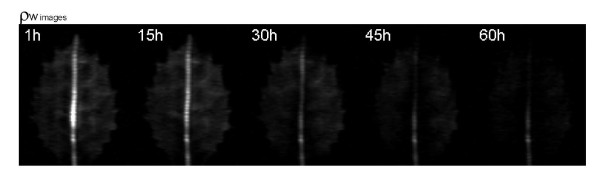
**^1^H density weighted Images (ρ_w_) of the last hours before the total loss signal in a *Quercus ilex *subjected to water deprivation for six weeks**.

The total loss of ρ_w _image signal occurred at the 9-10^th ^week in most leaves. The signal was lost first in the distal part of leaf, followed by loss in all parenchyma areas and finally in the middle and lower parts of the main leaf vein (Figure 6).

### T_2 _maps

**Figure 7:**
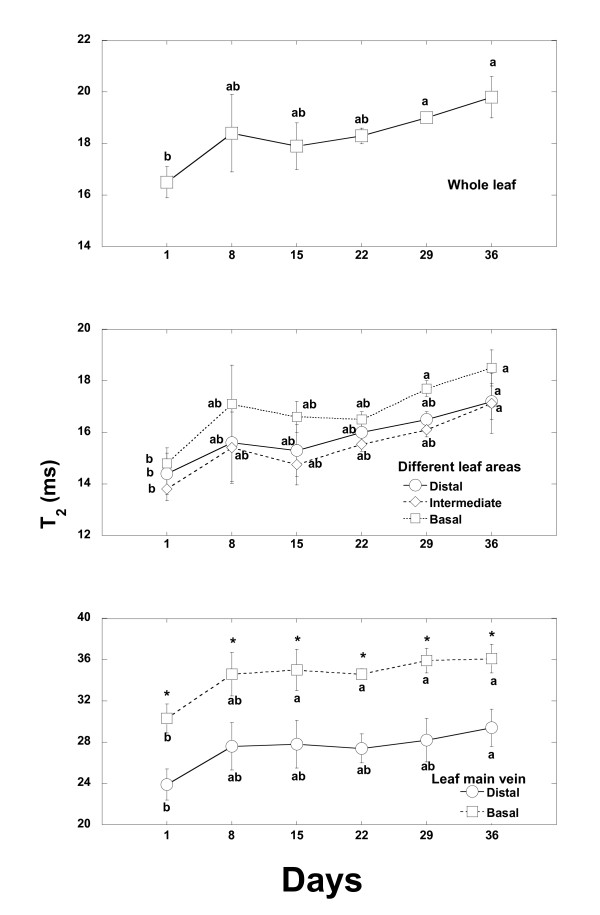
**Time course evolution of T_2 _(ms) values of different areas of the *Quercus ilex *leaf during the first five weeks after watering withdrawal**. The same letter means non significant differences, whereas different letters mean significant differences (p < 0.05) at different monitoring time.

Quantification of T_2 _weekly maps showed a tendency for T_2 _values to increase, although changes were statistically significant only after the fifth week of water deprivation. The increase was found in the whole leaf area and in most leaf tissues and areas studied and this rise was similar in the different leaf areas and tissues (Figure 7). No significantly different changes were observed in the ratios of T_2 _maps between different leaf zones and tissues (data not shown).

## Discussion

### Changes in mobile water content: ρ_w _images of the whole leaf area

The present results show that ρ_w _weighted MR images can be a useful tool to detect differing rates and patterns of water loss among the different tissues and parts of leaves. Due to the great variability between leaves (different plants, different leaf traits, forms and ages) the changes in water content at an hour to hour scale, which are less than 2% change, are difficult to detect when a set of leaf data is statistically processed, especially in advanced states of drought. However, these MRI techniques have shown to be a very useful tool to detect the changes in leaf water content and distribution at mid term periods of days and weeks. The observed changes in ρ_w _image intensities under progressive drought demonstrate that this technique, which has previously been used to detect patterns of water loss in other plant organs such as seeds [30] or bulbs [31] or in stems during cavitation and embolism [32,33], and whole leaves [18] can also be applied to study water loss in the different parts of the leaves.

Since the observed T_2 _values in the different leaf areas were relatively low (ranging from 13.8 ms to 36.0 ms), the presented proton density weighted images (acquired with a TE of 16 ms) may not be purely proton density weighted as they may have some T_2_-weighted contribution. This T_2_-weighted contribution will be stronger in regions with lower T_2 _values (like in the parenchyma). Nevertheless, in this work we are not comparing water content between the different leaf areas but changes of water content of the same leaf area over time. If no T_2 _changes were observed in the same leaf area over time, intensity changes on our ρ_w _images would still directly correspond to water content changes in that area even though they were not "purely" ρ_w _images. Conversely, we observed an increase on T_2 _values over the drought period in all the different leaf areas analyzed, which implies that there may be an underestimation on the water content losses that we are encountering.

The light application with an optical fiber did not have significant effects on patterns of ^1^H density changes. Probably the intensity of the light emitted by the optic fiber (4 ± 1 W m^-2^, PAR that is equivalent to 18.2 ± 4.5 μmol m^-2 ^s^-1^) was not enough to stimulate the leaf activity at a sufficient level to accelerate water losses, taking into account that the leaves of *Quercus ilex *in its natural environment are frequently submitted to high light intensities [27,34].

### Changes in water mobility: T_2 _maps

MRI of leaves at high magnetic field (B_0_) strengths can be difficult because of the presence of the many air spaces in the leaves. T_2 _decreases at higher field strengths due to the increase of field distortions and the presence of air spaces within a sample causes local susceptibility inhomogeneities which will also tend to decrease the observed T_2 _values [35]. Nevertheless, T_2 _values of the same sample measured at the same B_0 _field at different time points should not significantly vary unless there have been intrinsic changes in the sample (due to dryness in our particular study). Therefore, we can say that, in our study, T_2 _changes measured overtime are only dependent on leaf alterations and they are not a consequence of measuring T_2 _at high field.

T_2 _maps revealed an increase in the T_2 _transverse relaxation times of water within the leaf as drought progressed. The observed increase in T_2 _values with drought could therefore indicate a reduction of membrane permeability. A strong relation between cell membranes permeability and T_2 _has been previously described [8,36,37]. Water stored in vacuoles presents a greater T_2 _than water in the cytosol or water linked to macromolecules. Thus, T_2 _decays when membrane permeability increases and there is less water in the interior of the vacuole and more water linked to membrane structures. Although a decrease in T_2 _values could have been expected [30,38], in response to the observed decrease in water content (*ie. *in proton density), the contribution of membrane permeability decrease in increasing T_2 _values seem to have been greater than the proton density effect, overall resulting in an effective T_2 _increase. However, there are more possible reasons for changes in T_2 _throughout time such as ion solution changes, changes in leaf temperature, or veins cavitation. Increase of temperature has proved enhance T_2 _as response to leaf membrane injury when temperature increase up to 39-40°C [16,17]. However, these factors were unlikely to explain the increased T_2 _values because in our experiment soil substrate was the same throughout the experimental time and oak seedlings remained at 20-21°C during all the experiment. In fact, we observed T_2 _increase during the first week after last irrigation when the soil still remained wet and cavitation very probably had not occurred yet. Moreover, contrarily to the observed results vein cavitation during drought would have contributed decreasing the T_2 _values of the veins due to the increased air spaces [35].

The results also show that, in our study, ρ_w _images are more sensitive than T_2 _maps to detect changes in leaf water content during drought events. This may be due to T_2 _maps being acquired at a lower resolution than ρ_w _images and thus having more partial volume effects. But it can also be due to the opposing T_2 _changes produced by the structural damage and water content loss, and the need that one effect overcomes the other in order to generate absolute changes in T_2_. Our results show that significant changes of T_2 _are only observed significantly after 1 month of severe drought conditions, these results are consistent with the results observed in previous study where *Quercus ilex *in field conditions and under moderate drought conditions did not change significantly its T_2 _at whole leaf level [18]. All these results suggest a great capacity of *Quercus ilex *to resist drought conditions without changing its internal leaf water status, which only shows symptoms of change after severe drought conditions.

In freezing experiments, Millard *et al*., [39,40] described leaf T_2 _maps as a useful tool to detect the changes in the biophysical state of water during cold acclimation. The reduction of the level of vibration, rotation and translation energy during cold might produce several changes in spin-spin relaxation process. This does not occur during drought events without significant changes in temperature, such as in the present experiment. However, changes in T_2 _maps along time have also been shown to be sensitive and useful tools to detect water status changes in other plant tissues, for instance tubers during their development [41], or to study sap flow in stems [42], fruits after chemical treatments [43], and bulbs in storage [44].

### Changes in ρ_w _images and T_2 _maps in different leaf tissues

At the fifth week after the beginning of the drought experiment there was a greater water loss in the distal leaf area than in the areas near the petiole and in the main leaf vein than in parenchyma areas. Water concentrated more in the basal leaf zone and after continuous drought, necrosis of small areas of parenchyma was observed mainly in distal leaf areas. These dead zones increased in area with the passing of time under prolonged drought conditions. It should be noted that this flexible capacity to increase the proportion of water in parenchyma areas when the ^1^H density in the whole leaf area has diminished to 62% means that drought damage is delayed in the productive tissues. The accumulation of water in some areas of the leaf surface permitted those leaf areas to remain active. These results suggest a leaf capacity for internal water transport and readjustments among areas, mainly from main leaf vein to parenchyma and from distal to basal leaf areas together with an increase of water mobility that is very probably related to a decrease in cell membranes permeability.

## Conclusion

The results confirm that MRI studies offer a great potential to study the dynamics of water content changes among different leaf zones and tissues during changes in water availability. The results also show that foliar tissues begin necrosis when the ρ_w _image intensity decreases below 50% and that the distal leaf part loses the water faster when drought reaches a certain threshold (in our case 62% of water content with respect to the first MRI time point). All these results indicate a non homogeneous leaf response to stress with a differentiated capacity to mobilize water between its different parts and tissues. This provides important information about the different responses of the different leaf areas what should be taken into account when conducting physiological and metabolic drought stress studies.

## Methods

### Plant material and experimental design

3-year-old potted *Q. ilex *plants grown in a nursery (Forestal Catalana, S.A., Breda, Catalonia, Spain), and maintained for several weeks under Mediterranean ambient conditions outdoors, in the campus of the Autonomous University of Barcelona, (Barcelona, Catalonia, NE Spain, see Filella *et al*. [45] for a description of the site) were used in the study. They were grown in 2 L pots with a substrate composed of peat and sand (2:1), prior to being brought into the laboratory, where they were intensively irrigated for 5 days (to reach maximum substrate water containing capacity) to assure a maximum leaf water content. After those 5 days, the seedlings were submitted to water deprivation until death. We analyzed four leaves, each one from a different *Quercus ilex *seedling. In each case, the pot with the plant was introduced into the MRI spectrometer. First, a T_2 _map was acquired and then a series of ^1^H density weighted images were recorded each hour during the first 60 consecutive hours. This process was repeated every week at the same time for five successive weeks. This was considered the experimental period for all the leaves studied because at the end of this period all the leaves began to present symptoms of necrosis and some parts were dead. We used a blank solution of 99.8% deuterium-depleted water to standardize signal intensities on ρ_w _images among different weeks. In some cases, the measurements continued past these first five weeks, until the leaf was completely dead, in order to observe the final changes in leaf water content when the leaf became completely dry.

To detect possible effects of daily light hours on the changes in the patterns of water losses and on ρ_w _images and T_2 _maps, we built a system to provide light into the MRI magnet by using an optical fiber. In the 60 hour period in which the seedlings were inside the MRI scanner, the photoperiod was 12 h light:12 h darkness.

### MRI analyses

MRI measurements were carried out at 7 Teslas in a horizontal magnet (*BioSpec 70/30*, Bruker BioSpin, Ettlingen, Germany) equipped with actively shielded gradients (B-GA20S) using a quadrature 72 mm I.D. volume resonator. Intact leaves were positioned on a holder with a flat porous surface containing the reference blank solution (phantom) and only one coronal slice, containing the whole leaf thickness, was acquired. ρ_w _images were obtained using a fast spin echo sequence with the following imaging parameters: TR = 3000 ms, effective echo time (TE_eff_) = 16 ms, echo train length (ETL) = 4, field of view (FOV) = 10 × 5 cm^2^, acquisition matrix = 256 × 128 (thus, having an in plane resolution of 0.39 × 0.39 mm^2^), excited slice thickness = 1.4 cm, number of averages = 50, scan time per image = 1 h. T_2 _maps were acquired using a multi-echo sequence with parameters: TR = 3000 ms, 16 echoes with TE = 11-176 ms, field of view (FOV) = 15 × 5 cm^2^, acquisition matrix = 128 × 64 (thus, having an in plane resolution of 0.78 × 0.78 mm^2^), excited slice thickness = 1.4 cm, number of averages = 36, scan time per image = 1h 16min. T_2 _values were estimated, on a pixel by pixel basis, by fitting image intensities to a monoexponential decay using the MRI work-station software *ParaVision 4.0 *(Bruker BioSpin, Ettlingen, Germany). In order to have longitudinal quantitative information from the ρ_w _images, we used the blank solution intensity as the reference intensity. All pixel intensities were normalized to the blank and the percentage of water lost or water content, at different regions of interest, was always calculated with respect to the first MRI time point. Regions of interest were manually outlined on the ρ_w _images and then applied to the T_2 _maps. We selected approximately 10% of the leaf area situated closer to the petiole as basal area, a 10% of the central leaf area between the petiole and the distal area (the farthest one from the petiole) as intermediate leaf area, and a 10% of leaf area of the farthest zone from the petiole as distal leaf area. In all cases we avoided main and secondary leaf veins. In the case of main vein measurements we selected the central part of the main vein avoiding the border areas closer to parenchyma.

We analyzed the changes in ρ_w _images and T_2 _maps as regards the whole leaf area and in different leaf tissues. To study MRI sensitivity to detect changes over short time periods, we analyzed the changes in sets of 12 consecutive hours (with and without light application). To study the MRI sensitivity to detect changes at mid-term periods we analyzed the ρ_w _images and T_2 _maps every week until leaf death.

### Statistical analyses

To analyze the differences in ρ_w _images between hours and between weeks, we used the relative intensity with respect to the first hour as the dependent variable. This allows comparing the losses of water in each plant leaf with the course of time. We conducted a repeated measures ANOVA with different parts of the leaf area (basal, intermediate and distal) and different parts of the main leaf vein (basal and distal) as the independent factor followed by a Fisher post hoc test to detect the significance between different monitoring times.

To analyze the differences in spin-spin relaxation times (T_2_) between weeks and between different leaf areas, we used the T_2 _values as the dependent variable in the ANOVA test. This allows comparing the changes of T_2 _in each leaf area part with the course of time. We conducted repeated measures ANOVA with different parts of the leaf area (basal, intermediate and distal) and parts of the vein (basal and distal) as the independent factor followed by a Fisher post hoc test to detect the significance between different monitoring times. For all the statistical Analyses we used Statview 5.01 program (Abacus Concepts, SAS Institute Inc. Cary, NC, USA).

## Authors' contributions

JS and JP designed, performed and supervised the study and performed together with SL the experimental design and the data statistical analyses. SL established, conducted and controlled the RMI analyses. JS conducted the image analyses. All authors read and critically revised the manuscript.
